# Role of SiN*_x_* Barrier Layer on the Performances of Polyimide Ga_2_O_3_-doped ZnO p-i-n Hydrogenated Amorphous Silicon Thin Film Solar Cells

**DOI:** 10.3390/ma7020948

**Published:** 2014-02-07

**Authors:** Fang-Hsing Wang, Hsin-Hui Kuo, Cheng-Fu Yang, Min-Chu Liu

**Affiliations:** 1 Department of Electrical Engineering, and Graduate Institute of Optoelectronic Engineering, National Chung Hsing University, Taichung 40227, Taiwan; E-Mails: fansen@dragon.nchu.edu.tw (F.-H.W.); stmike18@yahoo.com.tw (M.-C.L.); 2 Department of Electrical Engineering, National University of Kaohsiung, Kaohsiung 81148, Taiwan; E-Mail: hhkuo@nuk.edu.tw; 3 Department of Chemical and Materials Engineering, National University of Kaohsiung, Kaohsiung 81148, Taiwan

**Keywords:** silicon nitride (SiN*_x_*), polyimide (PI), PECVD, GZO thin film, silicon thin film solar cell

## Abstract

In this study, silicon nitride (SiN*_x_*) thin films were deposited on polyimide (PI) substrates as barrier layers by a plasma enhanced chemical vapor deposition (PECVD) system. The gallium-doped zinc oxide (GZO) thin films were deposited on PI and SiN*_x_*/PI substrates at room temperature (RT), 100 and 200 °C by radio frequency (RF) magnetron sputtering. The thicknesses of the GZO and SiN*_x_* thin films were controlled at around 160 ± 12 nm and 150 ± 10 nm, respectively. The optimal deposition parameters for the SiN*_x_* thin films were a working pressure of 800 × 10^−3^ Torr, a deposition power of 20 W, a deposition temperature of 200 °C, and gas flowing rates of SiH_4_ = 20 sccm and NH_3_ = 210 sccm, respectively. For the GZO/PI and GZO-SiN*_x_*/PI structures we had found that the GZO thin films deposited at 100 and 200 °C had higher crystallinity, higher electron mobility, larger carrier concentration, smaller resistivity, and higher optical transmittance ratio. For that, the GZO thin films deposited at 100 and 200 °C on PI and SiN*_x_*/PI substrates with thickness of ~000 nm were used to fabricate p-i-n hydrogenated amorphous silicon (α-Si) thin film solar cells. 0.5% HCl solution was used to etch the surfaces of the GZO/PI and GZO-SiN*_x_*/PI substrates. Finally, PECVD system was used to deposit α-Si thin film onto the etched surfaces of the GZO/PI and GZO-SiN*_x_*/PI substrates to fabricate α-Si thin film solar cells, and the solar cells’ properties were also investigated. We had found that substrates to get the optimally solar cells’ efficiency were 200 °C-deposited GZO-SiN*_x_*/PI.

## 1. Introduction

Transparent conducting oxides (TCOs) are electrical conductive materials with a comparably low absorption of light. TCOs also show a good combination of electrical conductivity at ambient temperature and optical transparency in a visible region. They are usually prepared with thin film technologies and widely used in the applications of the various opto-electrical devices such as solar cells, flat panel displays (FPDs), opto-electrical interfaces, and circuitries [1]. Thus, as n-type TCOs are of special importance for thin film solar cell production, indium-tin oxide (ITO) [2] and the reasonably priced aluminum-doped zinc oxide (ZnO:Al) [3] are discussed with view on preparation, characterization, and special occurrences. Raniero *et al*. [4] used a plasma enhanced chemical vapor deposition (PECVD) system with a single chamber to study the influence of hydrogen plasma on the ITO thin films. They prove that the optical transmittance for the ITO thin films decrease to 15% and 19%, respectively, when using power densities of 47 and 80 mW/cm^2^, during the first 60 s of plasma exposition. In addition to instability to hydrogen plasma, ITO also exhibits other disadvantages including toxicity and increasing price due to the global indium shortage. Accordingly indium-free TCO materials have attracted considerable attention from many researchers. Although Al-doped ZnO thin films present favorable electrical properties, aluminum exhibits a significantly higher reactivity to oxygen, which leads to oxidation during thin film growth and then results in the degradation of the electrical properties. Because gallium is less receptive to oxidation, Ga-doped ZnO (GZO) TCO materials have been reported to have a better stability [5,6].

Recently, the necessity of studying the deposition process of TCO thin films on polymer substrates has increased, as the polymer substrates are suitable for FPDs and optoelectronics [6–8]. As the polymer substrates are cheaper, lighter, and more flexible compared to conventional glass substrate, they could be effectively used in applications such as flexible display and flexible solar cells. However, polymer substrates exhibit several demerits such as poor thermal, optical, and electrical properties. The difference in refractive indices of used substrates and TCO electrodes causes optical reflection in thin film solar cells, which results in lower absorption of light for devices. In the past, an anti-reflection layer between substrates (glass or polymer) and TCO has been used to reduce the loss of light due to optical reflection. Besides improving surface energy and adhesion between TCO and polymer substrate, oxide buffer layers or barrier layers are typically used. Ahn *et al*. [9] deposited SiO*_x_* buffer layers at various oxygen pressures to investigate the effect of oxygen pressure of SiO*_x_* buffer layers on the electrical properties of the GZO thin films, which were deposited on poly-ethylene telephthalate (PET) substrates. They found that with increasing oxygen pressure during the deposition of SiO*_x_* buffer layers, the electrical resistivity of the GZO-SiO*_x_* bi-layer thin films on PET substrates gradually decreased from 7.6 × 10^−3^ to 6.8 × 10^−4^ Ω·cm, due to the enhanced mobility of GZO thin films.

Raniero *et al*. [10] added the buffer layer in p-i-n thin film solar cells to form a TCO coated glass/p-α-SiC:H/buffer/i-(nc-Si/α-Si:H)/n-α-Si:H/Ag structure. They used deposition time to control for the thickness of buffer layer and found that *V*_oc_ increased almost linearly with the buffer layer thickness up to a thickness equivalent to 10 s. On the other hand, they also found that *J*_sc_ stabilized at around 20 mA/cm^2^, as the buffer layers’ deposited time was between 5 and 10 s. Pei *et al*. [11] showed that the AZO thin films deposited on Al_2_O_3_-buffered flexible substrates showed a significant decrease of sheet resistance when compared with those deposited on bare polymer. Araujo *et al*. [12] added a disperse carbon interlayer between the n-α-Si:H layer and an aluminum zinc oxide (AZO) back contact. They have found that an α-Si:H tandem solar with this structure could have a 10% increase in the short current density (*J*_sc_) and a 20% increase in the efficiency compared to a standard solar cell. In this work, the properties of the GZO thin films grown on polyimide (PI) substrates by RF sputtering processing under different deposition temperatures (room temperature-RT, 100 and 200 °C) were first studied. In the past, silicon nitride (SiN*_x_*) thin films had shown anti-reflection property and could be used to improve the efficiency of the Si-based solar cells [13–15]. The optimization of the refractive index of the SiN*_x_* thin films in the visible light region to achieve a value between the refractive index of glass (1.5) and TCO film (2.0), and the refractive index of SiN*_x_* thin films can be engineered by changing the silicon or nitrogen content in the thin films. The SiN*_x_* thin films were also grown on the p-Si(100) substrate by transformer coupled plasma chemical vapor deposition (TCP-CVD) [16]. The deposited SiN*_x_* thin films have good properties and advantages as low temperature processed barrier layers, and they can be adopted in the thin film transistor (TFT) type ferroelectric random access memory (FRAM).

These results prove that the additions of oxide buffer layers or barrier layers will improve the efficiencies of the fabricated thin film solar cells and FRAM. In this study, the amorphous thin SiN*_x_* barrier layers were deposited by the PECVD method on the PI substrates before the GZO thin films were deposited. The GZO thin films deposited on SiN*_x_* thin films to form a bi-layer structure were compared with GZO thin films without the SiN*_x_* barrier layers. X-ray diffraction (XRD) pattern, surface morphology observations, hall measurements, and optical transmittance ratio were used to monitor the changes in the structural, the electrical, and the optical properties of the GZO thin films deposited on PI with SiN*_x_* and without the SiN*_x_* barrier layers. Thin film solar cells using hydrogenated amorphous Si (α-Si:H) and nanocrystalline Si (nc-Si:H) are among the most well-developed thin film photovoltaic materials. Finally, the GZO thin films were also deposited at 100 and 200 °C on PI and SiN*_x_*/PI substrates to form the GZO/PI and GZO-SiN*_x_*/PI structures for the fabrication of the α-Si:H thin-film solar cells. The surfaces of GZO/PI and GZO-SiN*_x_*/PI structures were etched by 0.5% HCl solution in order to increase the haze ratio, and the α-Si:H thin-film solar cells were fabricated on the etched GZO/PI and GZO-SiN*_x_*/PI structures, and their I–V properties were also investigated.

## 2. Experimental Details

**Figure 1.**
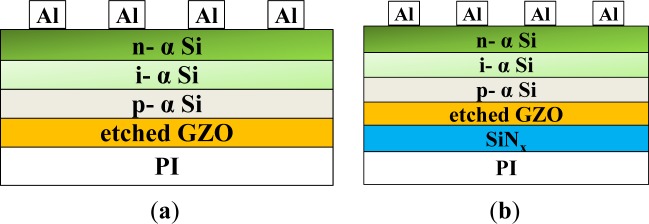
Structure configurations of the two different hydrogenated amorphous silicon thin film solar cells, fabricated on (**a**) etched gallium-doped zinc oxide/polyimide (GZO/PI) substrates and (**b**) etched GZO-silicon nitride (SiN*_x_*_)_/PI substrates.

In this work, the RF (13.56 MHz) magnetron sputtering process was used to deposit the GZO thin films. ZnO (97 wt%, 5N, Admat Inc., Norristown, PA, USA) doped with Ga_2_O_3_ (3 wt%, 5N, Admat Inc., Norristown, PA, USA) was mixed, ground, calcined at 1000 °C for 5 h, and sintered at 1400 °C to form the ceramic targets with 2-inches in diameter. The used substrate was 33 mm × 33 mm × 2 mm polyimide (abbreviated as PI) (Taimide Tech. Inc., Hsinchu county, Taiwan), and the substrates with two different structures were used to fabricated hydrogenated α-Si:H solar cells. For the first one, only the PI was used as the substrate (Figure 1a) and for the second, SiN*_x_* was chosen as a barrier layer between the GZO and PI substrates (Figure 1b). As the glass transferring temperature of PI was higher than 400 °C and it did not decompose in air and in N_2_, the temperature chosen was also higher than 400 °C. Based on this, even when 200 °C was used as deposition temperature, the PI substrates were not expected to have any variations in their properties. SiN*_x_* thin films were fabricated using a single-chamber plasma-enhanced chemical vapor deposition (PECVD) unit. Thicknesses of the SiN*_x_* and GZO thin films were one of the most important parameters to influence the characteristics of the superstrate p-i-n α-Si:H thin film solar cells. For this reason, thicknesses of the SiN*_x_* and GZO thin films were measured using a SEMF-10 ellipsometer (Nano-view, Hanyang, Korea) and confirmed by field emission scanning electron microscopy (FESEM) (JEOL JSM-6700, Tokyo, Japan). Deposition rates and thin films’ thicknesses of the SiN*_x_* and GZO thin films were determined by averaging five data sets obtained by FESEM.

Before the deposition process was started, the base chamber pressure of the sputtering system was pumped to less than 1 × 10^−6^ Torr, then the deposition parameters were controlled at different pressures and powers. The optimal deposition parameters were a RF power of 50 W and a working pressure of 5 × 10^−3^ Torr because the deposited GZO thin films had the flattest surface and the most acceptable deposition rate. The GZO thin films were also deposited at different temperatures, where room temperature (RT), 100 and 200 °C were used. The thicknesses of the GZO and SiN*_x_* thin films were 160 ± 12 nm and 150 ± 10 nm when controlling the deposition time. The electrical properties of the GZO and bi-layer GZO-SiN*_x_* thin films were determined by a Hall effect measurement, while the thin films’ crystalline structures were identified by X-ray diffraction (XRD) (Bruker, Billerica, MA, USA). Optical transmittances of the GZO and bi-layer GZO-SiN*_x_* thin films on PI substrates were measured by using a ultraviolet-visible spectroscopy (UV-Vis) spectrophotometer (Hitachi U3300, Kenichi Sato, Japan). After the physical and electrical properties of the GZO and bi-layer GZO-SiN*_x_* thin films were measured, the GZO thin films with a thickness of 1000 nm were deposited on PI and SiN*_x_*/PI substrates. After that, the surfaces of the GZO thin films were etched by wet etching performed in diluted HCl solution with concentrations of 0.5% in H_2_O to acquire the textured GZO thin films. The thickness of the etched GZO thin films was around 650 nm, which was obtained by controlling the etched time.

Superstrate p-i-n α-Si:H thin film solar cells were also fabricated using a single-chamber PECVD unit at 200 °C on the etched GZO-PI and GZO/SiN*_x_*-PI substrates, as Figure 1 shows. The working pressure was 700 × 10^−3^ Torr and the deposition power was 20 W. The p-type α-Si (thickness was about 20 nm) was deposited by controlling the gas flow rates of H_2_ = 100 sccm, SiH_4_ = 20 sccm, CH_4_ = 10 sccm, and B_2_H_6_ = 40 sccm; The i-type α-Si (about 400 nm) was deposited by controlling the gas flow rates of H_2_ = 100 sccm and SiH_4_ = 10 sccm; The p-type α-Si (about 50 nm) was deposited by controlling the gas flow rates of H_2_ = 100 sccm, SiH_4_ = 20 sccm, and PH_3_ = 20 sccm, respectively. The desired thicknesses of all thin films were obtained by controlling deposition time. The current-voltage characteristic of the fabricated solar cells was measured under an illumination intensity of 300 mW/cm^2^ and an AM 1.5 G spectrum, and all measurements were performed at room temperature.

## 3. Results and Discussion

**Figure 2.**
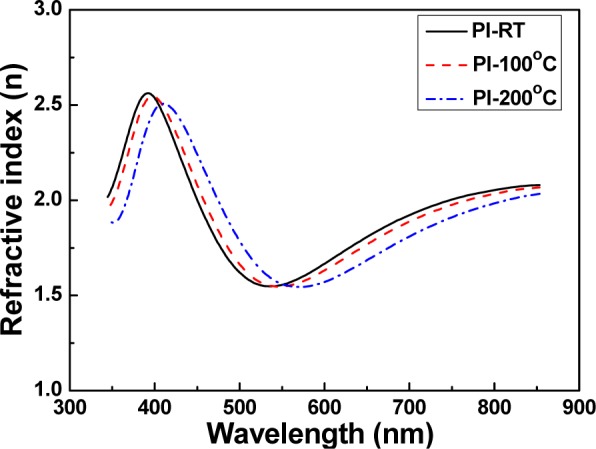
Refractive index of the GZO thin films as a function of deposition temperature.

When the SiN*_x_* thin films are used as barrier layers between the GZO thin films and PI substrates, the refractive index of the SiN*_x_* thin films have to match that of the GZO thin films. For that, the refractive index of the GZO thin films was first measured as a function of deposition temperature. As Figure 2 shows, as the deposition temperatures were RT, 100 and 200 °C, the maximum values of the refractive index were 2.5618, 2.5466, and 2.5064, respectively, and the wavelengths to reveal the maximum index value were 391.5, 396.9, and 409.0 nm, respectively. The maximum refractive index decreased and the wavelength to reveal the maximum index was shifted to a lower value as the deposition temperature of the GZO thin films was raised.

**Figure 3.**
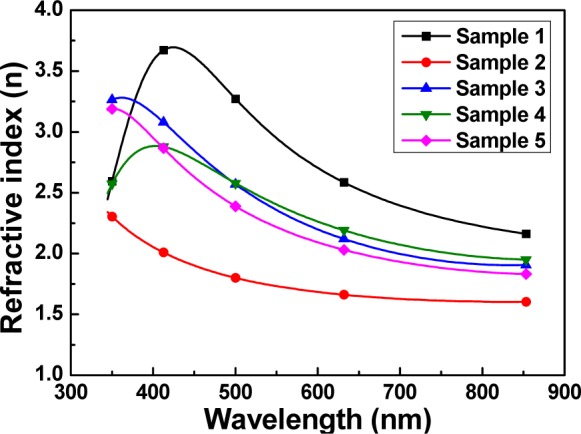
Refractive index of the SiN*_x_* thin films as a function of deposition parameters.

In this study, SiN*_x_* thin films were deposited by PECVD system by changing silane (SiH_4_) and ammonia (NH_3_) flow rates. Table 1 shows the five PECVD deposition parameters of the SiN*_x_* thin films. The refractive indexes of the SiN*_x_* thin films as a function of deposition parameters and optical wavelength are shown in Figure 3. The main composite of SiN*_x_* is Si_3_N_4_, which has a refractive index of about 2.05. However, the refractive index of the SiN*_x_* thin films is dependent on the *x* value. The optimal deposition parameters for the SiN*_x_* thin films used in this study were a working pressure of 800 × 10^−3^ Torr, a deposition power of 20 W, a deposition temperature of 200 °C, and gas flow rates of SiH_4_ = 20 sccm and NH_3_ = 210 sccm, respectively. The SiN*_x_* thin films deposited at those parameters had a refractive index smaller than 2.5 at the wavelength of 350–850 nm.

**Figure 4.**
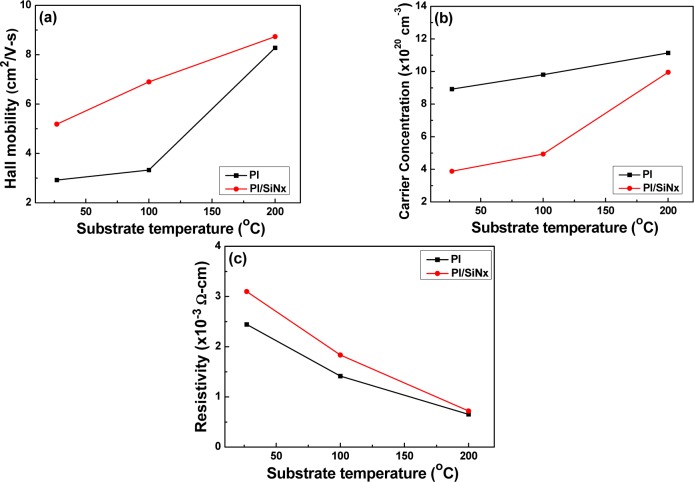
Variations of the (**a**) Hall mobility; (**b**) carrier concentration; and (**c**) resistivity as a function of the GZO thin films deposited on different substrates as a function of deposition temperature.

Figure 4 shows the variations of the carrier concentration, carrier mobility, and resistivity of the GZO thin films deposited by radio frequency (rf) magnetron sputtering at different deposition temperatures on PI and SiN*_x_*/PI substrates. Figure 4a shows that the carrier mobility of the GZO thin films increased with raising deposition temperature and was independent of the used substrates. Figure 4a also shows that as the SiN*_x_* was used as the barrier layer, the carrier mobility of the GZO thin films apparently improved. As the deposition temperature was raised from RT to 200 °C, the carrier mobility increased from 2.92 to 8.28 cm^2^/V-s for PI substrates and from 5.19 to 8.73 cm^2^/V-s for SiN*_x_*/PI substrates. Figure 4b shows that the carrier concentration of the GZO thin films also increased with raising deposition temperature. When the GZO thin films are deposited on PI or SiN*_x_*/PI substrates using the RF sputtering process, many defects result, which inhibit electron movement. Using higher deposition temperatures during the deposition process can lead to an enhancement of the thin films’ densification and crystallization. That is a reason to decrease the numbers of defects and pores in the GZO thin films and to increase in the inhibition of barrier electron transportation [17]. Also, as the deposition temperature was raised from RT to 200 °C, the carrier concentration increased from 8.92 × 10^20^ to 11.1 × 10^20^ cm^−3^ for PI substrates and from 3.88 × 10^20^ to 9.95 × 10^20^ cm^−3^ for SiN*_x_*/PI substrates.

Figure 4c shows the dependences of resistivity of the GZO thin films on deposition temperatures and used substrates. In this study, the carrier mobility (Figure 4a) and carrier concentration (Figure 4b) increased with raising deposition temperature and reached a maximum at 200 °C. Resistivity of the GZO thin films is proportional to the reciprocal value of the product of the carrier concentration N and the mobility μ:

$$\rho =\frac{1}{\text{Ne}\times \mu }$$ (1)

As Equation (1) shows, both the carrier concentration and the carrier mobility contribute to the resistivity. The minimum resistivity of the GZO thin films at a deposition temperature of 200 °C is mainly influenced by both the carrier concentration and the carrier mobility being at their maximum. As the deposition temperature was raised from RT to 200 °C and PI and SiN*_x_*/PI were used as substrates, the resistivity decreased from 2.44 × 10^−3^ to 0.651 × 10^−3^ Ω·cm for PI substrate and from 31.0 × 10^−3^ to 0.718 ×10^−3^ Ω·cm for SiN*_x_*/PI substrate. In order to match the SiN*_x_* thin films’ refractive indexes with those of the GZO thin films, the RT-deposited GZO thin films are not suitable, but the 100 °C- and 200 °C-deposited GZO thin films are suitable for further application in the fabrication of thin film solar cells. The GZO thin films deposited at RT showed an amorphous phase and no observable crystalline phase, which is the second reason why the RT-deposited GZO thin films are not used for further application. From the results shown in Figure 4, the RT-deposited GZO it can be seen that the thin films had the smallest carrier concentration and mobility and the largest resistivity, which lead to the third reason for not using the RT-deposited GZO thin films for further applications.

**Figure 5.**
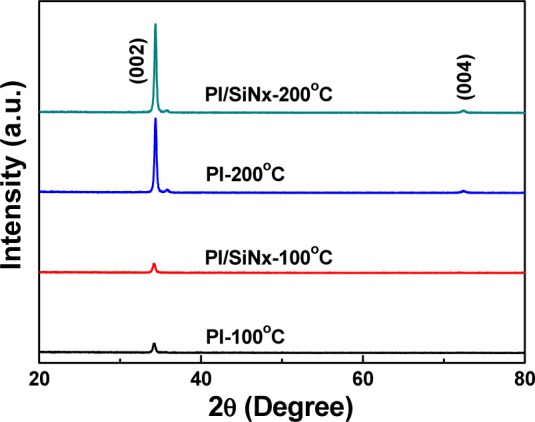
X-ray diffraction (XRD) diffraction patterns of the GZO thin films deposited on different substrates as a function of deposition temperature.

The XRD patterns of the GZO thin films deposited on the PI and SiN*_x_*/PI substrates and at deposition temperatures of 100 and 200 °C were investigated, and the results are compared in Figure 5, in which the (002) peaks of all the GZO thin films are exhibited. The diffraction intensity of the (002) peak critically increased as the deposition temperature increased from 100 to 200 °C. The 200 °C-deposited GZO thin films exhibited the (004) peak, and both results were independent of the used substrates (PI or SiN*_x_*/PI). The (002) peaks of the GZO thin films prepared under the structures of the 100 °C-deposited GZO-PI (abbreviated as Substrate A), 100 °C-deposited GZO-SiN*_x_*/PI (Substrate B), 200 °C-deposited GZO-PI (Substrate C), and 200 °C-deposited GZO-SiN*_x_*/PI (Substrate D) were situated at 2θ = 34.14°, 34.16°, 34.36°, and 34.36°, respectively. The lattice constant *c* was calculated by using the 2θ value, the calculated lattice constants (*c*), which are compared in Table 2, were 0.5249, 0.5246, 0.5210, and 0.5210, respectively, as GZO thin films were deposited on Substrate A, Substrate B, Substrate C, and Substrate D, respectively. All the calculated lattice constants *c* of the GZO thin films smaller than that of the ZnO thin films are considerable, because the radius of Ga^3+^ ions (62 pm) is smaller than that of Zn^2+^ ions (72 pm).

As Figure 5 shows, the full width at half maximum (FWHM) values for the (002) peak of the GZO thin films ranged from 0.360, 0.378, 0.296, to 0.293, as Substrate A, Substrate B, Substrate C, and Substrate D were used, as Table 2 shows. Those results suggest that the deposition temperature, rather than the used substrate, is the most important parameter influencing the crystalline structure. These results also suggest that GZO thin films deposited at higher deposition temperatures have a better crystalline structure and that the defects in the GZO thin films decrease with raising deposition temperature. Because the reason for this is that when a higher temperature is used to deposit the GZO thin films, GZO particles have a higher active energy for molecular adhesion to the substrates, which improves crystallization and decreases the number of defects of the thin films, which in turn leads to a decrease of the FWHM value. The results in Figure 4 show that, as the deposition temperature is raised from RT to 200 °C, a more uniform *c*-axis orientation is obtained in the 200 °C-deposited GZO thin films. Thus, better crystallinity resulting from a stronger *c*-axis orientation could be achieved for Substrate C and Substrate D. The results in Figures 4 and 5 show that the increase of both the carrier concentration and carrier mobility with raising deposition temperatures is attributed to the enhancement in the crystallinity of the GZO thin films.

Determination of the optical band gap (*E*_g_) is often necessary to develop the electronic band structure of a thin-film material. When the extrapolation method is used, the *E*_g_ values of the GZO thin films can be determined from the absorption edge for direct interband transition, which can be calculated using the relation in Equation (2):

$${\left(\alpha hv\right)}^{2}=c\;(hv-E_{g})$$ (2)

where α is the optical absorption coefficient, *c* is the constant for direct transition, *h* is Planck’s constant, and *ν* is the frequency of the incident photon [18]. The *E*_g_ values can be found at the point where (α*hv*)^2^ is zero and the linear dependence of (α*hv*)^2^ on *hν* indicates that the GZO thin films are direct transition type semiconductors.

**Figure 6.**
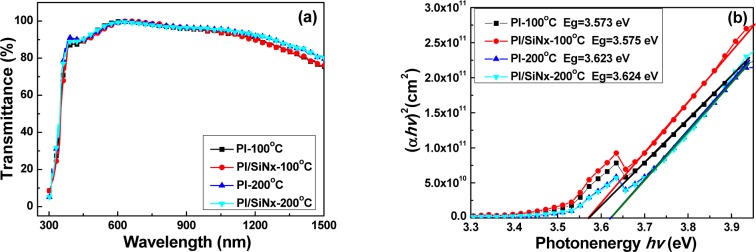
(**a**) Transmittance and (**b**) (α*hv*)^2^ *vs*. *hν−E*_g_ plots of the GZO thin films deposited on different substrates as a function of deposition temperature.

The transmission ratios of the GZO thin films deposited on Substrate A, Substrate B, Substrate C, and Substrate D plotted against wavelengths in the region of 300–1500 nm were measured, and the results are shown in Figure 6a. As the GZO thin films were deposited on Substrate A and substrate B, the optical transmission rate in the visible region of 400–700 nm is higher than 94% and has a maximum of 95.6%, and in the near infrared region of 700–1400 nm is higher than 89% for all the thin films regardless of the deposition temperature; As the GZO thin films were deposited on Substrate B and Substrate D, the optical transmission rate in the visible region of 400–700 nm is also higher than 93.5% and has a maximum of 95.0%, and in the near infrared region of 700–1400 nm it is higher than 84%. These results show that the SiN*_x_* layer does not degenerate the transmission ratio of the GZO thin films on PI substrates. In the transmission spectra of Substrate A, Substrate B, Substrate C, and Substrate D, the optical band edge shows no apparent shift. This result suggests that the *E*_g_ value is independent of the used substrates (the PI or SiN*_x_*/PI) and that it is influenced by the deposition temperature. Independently of which PI or SiN*_x_*/PI was used as substrate and whether 100 or 200 °C was used as the deposition temperature, a greater sharpness is noticeable in the curves of the absorption edge, as can be seen in Figure 6a.

Figure 6b plots (α*hv*)^2^ against *hv* (energy) in accordance with Equation (1), and the *E*_g_ values can be found by extrapolating a straight line at (α*hv*)^2^ = 0. The calculated *E*_g_ values of the GZO thin films as a function of deposition temperature are also shown. As Table 2 and Figure 6b show, the *E*_g_ values were 3.573, 3.575, 3.623, and 3.624 eV, respectively, for the deposition parameter, and the substrates for the GZO thin films were Substrate A, Substrate B, Substrate C, and Substrate D. These results suggest that as the SiN*_x_* thin films are used as the barrier layers between the PI and GZO thin films, they have almost no influence on the *E*_g_ value of the GZO (or bi-layer GZO-SiN*_x_*) thin films. The results in Figure 2 and Figure 6b suggest again that the deposition temperature is the important factor that influences the crystallization and then will influence the *E*_g_ value independent of the fact whether the barrier layer (SiN*_x_*) is used or not. As Figure 6a shows, the blue-shift in the absorption edge of the GZO thin films was not observed and it could be obtained from the calculated *E*_g_ values shown in Figure 6b. This blue-shift can be explained by the Burstein-Moss shift, a shift of the Fermi level into the conduction band, which enhances the optical *E*_g_ value by the energy, as follows [19,20]:

$$\Delta E_{g}^{BM}=\frac{\hbar^{2}k_{F}^{2}}{2}\left(\frac{1}{m_{e}}+\frac{1}{m_{h}}\right)=\frac{\hbar^{2}k_{F}^{2}}{2m_{vc}^{*}}$$ (3)

where *k_F_* stands for the Fermi wave vector and is given by *k_F_* *=* (3π^2^*n_e_*)^1/3^, *m_e_* is the effective mass of electrons in the conduction band, and *m_h_* is the effective mass of holes in the valence band, which can be simplified as m^*^_vc_, the reduced effective mass. Δ*E*_g_ value can be rewritten by inducing *k_F_* for the carrier concentration *n_e_*:

$$\Delta E_{g}^{BM}=\frac{\hbar^{2}}{2m_{vc}^{*}}{\left(3\pi^{2}n_{e}\right)}^{2/3}$$ (4)

Equation (4) shows that the Burstein-Moss shift of the *E*_g_ value to the larger value is due to the increase in carrier concentration (*n_e_*).

**Figure 7.**
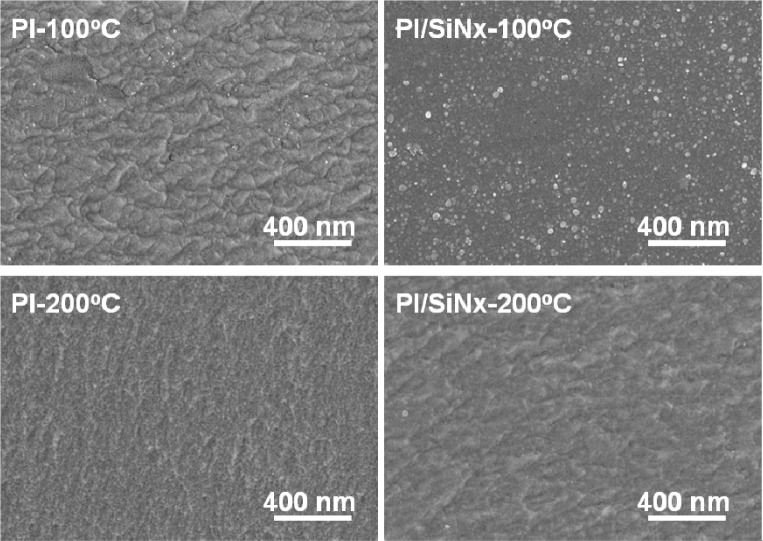
Surface morphologies of the un-etched GZO thin films deposited on different substrates as a function of deposition temperature.

Morphologies of the GZO thin films deposited on different substrates and at different temperatures are shown in Figure 7, which indicates that as the substrate and deposition temperature are changed, the surface morphologies are apparently changed as well. When deposited at RT (not shown here), the morphology of the GZO thin films exhibited a flat surface and no grain growth or particle aggregation was observed. As the deposition temperature was increased to 100 and 200 °C, a nano-crystalline structure of the GZO grains was observed, as is shown in Figure 7. However, the variations of crystallization sizes are dependent on the deposition temperature and the used substrates, and they are not easily calculated from the observation of the surface morphology. We will illustrate the variations of grain sizes from the XRD patterns and Equation (5) [21]:

$$D=\frac{0.9\lambda }{\beta \text{con}(\theta )}$$ (5)

As Figure 7 and Table 2 show, the average crystallization sizes were 31.8, 27.0, 35.2, and 28.0 nm, respectively, when Substrate A, Substrate B, Substrate C, and Substrate D, were used to deposit the GZO thin films. This is caused by the fact that with an increase in deposition temperature from 100 to 200 °C—the higher deposition temperature, the better the crystallinity of the GZO thin films (Figure 5)—the plasma GZO molecules do not get enough energy to improve the grain growth. Rough interfaces are usually introduced into solar cells by using substrates with a textured surface [22,23]. For solar cells in a superstrate configuration, usually a glass plate covered with textured transparent conductive oxide (TCO) thin film is used. The TCO layer forms the front contact of the superstrate solar cell and has to exhibit good electrical (high conductivity) and optical (high transmittance) properties. A suitable textured surface is very important to scatter an incident light, particularly long wavelength light (red and near-infrared), to extend the effective path length within the active silicon layer and subsequent light trapping inside the absorber material of the solar cell [22,23]. In this study, diluted a HCl etching of the GZO thin films was carried out at room temperature to develop the textured surface for enhancing the efficiency of solar cells.

**Figure 8.**
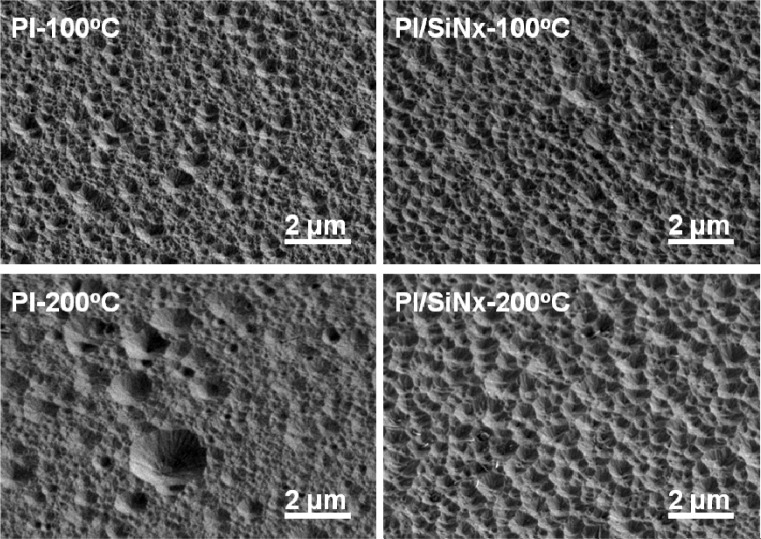
Surface morphologies of the etched GZO thin films deposited on different substrates as a function of deposition temperature.

Figure 8 shows the surface morphologies of the etched GZO thin films. The etched time was about 30 s. Comparing the results shown in Figures 7 and 8, the surface roughness increased significantly after HCl etching and the etching process caused the thin films’ surfaces to develop a crater-like structure. As the etched results for the GZO thin films with and without SiN*_x_* barrier layer are compared, the surface images of the films with barrier layer have more uniform cave sizes than those of films without barrier layer. The haze ratio of the thin films before and after HCl etching was measured using a haze meter (Nippon Denshoku, NDH 2000, Saitama, Japan). However, the haze ratios of the non-etching Substrate C and Substrate D were 0.37% and 0.48%, respectively. As the etching process was used, the haze ratios of Substrate A, Substrate B, Substrate C, and Substrate D were 12.6%, 14.0%, 18.9%, and 37.3%, respectively. Figure 8 reveals that the etched GZO thin films will effectively scatter an incident light and enhance light trapping inside the absorber material of the Si thin film solar cells.

**Figure 9.**
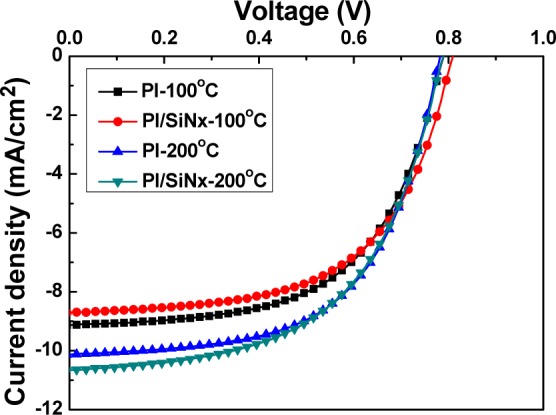
Current-voltage characteristics of the p-i-n α-Si:H thin-film solar cells under illumination.

Superstrate p-i-n hydrogenated α-Si thin film solar cells were fabricated using a single-chamber PECVD unit at 200 °C. The structures of the designed solar cells are shown in Figure 1. No antireflective coatings were deposited on the cells. Figure 9 shows the measured current–voltage characteristics of the solar cells (substrate size 3.3 × 3.3 cm^2^) under illumination. The values of open-circuit voltage (*V*_oc_), short-circuit current density (*J*_sc_), fill factor (*F.F.*), and efficiency (η) are measured for the devices fabricated on the etching substrate. As the etched Substrate A, Substrate B, Substrate C, and Substrate D were used to fabricate the thin-film silicon solar cells, the *V*_oc_ values of the solar cells were 0.790, 0.805, 0.785, and 0.790 V, the *J*_sc_ values were 9.13, 8.753, 10.13 mA/cm^2^, and 10.64 mA/cm^2^. The *F.F.* values were 0.580, 0.565, 0.588, and 0.553, and the efficiencies were 4.20 ± 0.19, 3.96 ± 0.10, 4.65 ± 0.10, and 4.79 ± 0.15, respectively. As Figure 9 shows, the *V*_oc_ value had no apparent trend, the *J*_sc_ value increased, and the *F.F.* value decreased with raising deposition temperature. These measured results of the fabricated α-Si thin film solar cells are also compared in Table 2. The greater efficiencies when using the 200 °C-PI and 200 °C-SiN*_x_*/PI substrates are mainly ascribable to the fact that as the haze ratio increases, the absorption of light increases, and as a consequence, the short-circuit current density increases. Another reason for the 200 °C-SiN*_x_*/PI substrates having a greater efficiency is that the antireflection (AR) coating effect of buffer SiN*_x_* thin films is deposited before the thin-film α-Si solar cells are produced in order to obtain an ideal broadband AR property in the visible and near-infrared wavelength range [13].

## 4. Conclusions

For the GZO thin films, as their deposition temperature was raised from RT to 200 °C, the carrier mobility and carrier concentration increased and the resistivity decreased independently of the used substrates. As GZO thin films were deposited at 100 and 200 °C on PI and SiN*_x_*/PI substrates, the optical transmission rate in the visible region of 400–700 nm was higher than 93.5% and had a maximum of 95.6%, and in the near infrared region of 700–1400 nm it was higher than 84%. As the deposition temperature of the GZO thin films was raised from RT to 200 °C, the carrier mobility increased from 2.92 to 8.28 cm^2^/V-s for PI substrates and from 5.19 to 8.73 cm^2^/V-s for SiN*_x_*/PI substrates, the carrier concentration increased from 8.92 × 10^20^ to 11.1 × 10^20^ cm^−3^ for PI substrates and from 3.88 × 10^20^ to 9.95 × 10^20^ cm^−3^ for SiN*_x_*/PI substrates, the resistivity decreased from 2.44 × 10^−3^ to 0.651 ×10^−3^ Ω-cm for PI and SiN*_x_*/PI substrate and from 31.0 × 10^−3^ to 0.718 ×10^−3^ Ω-cm for SiN*_x_*/PI substrate, respectively. As the GZO thin films were deposited on Substrate A, Substrate B, Substrate C, and Substrate D, the *E*_g_ values were 3.573, 3.575, 3.623, and 3.624 eV and the average crystallization sizes were 31.8, 27.0, 35.2, and 28.0 nm, respectively. The haze ratio of the etched GZO thin films increased from 12.6% (14.0% with SiN*_x_* barrier layer) to 18.9% (37.3%) as the deposition temperature was raised from 100 to 200 °C. The efficiency of the fabricated p-i-n hydrogenated amorphous silicon thin film solar cells increased from 4.20 ± 0.19 (3.96 ± 0.10 with SiN*_x_* barrier layer) to 4.65 ± 0.10 (4.79 ± 0.15) as the deposition temperature was raised from 100 to 200 °C. This study shows that the higher deposition temperature of the GZO thin films and the use of SiN*_x_* as the barrier layer are two important technologies to improve the efficiency of fabricated p-i-n amorphous silicon thin film solar cells.

## Figures and Tables

**Table 1.** Plasma enhanced chemical vapor deposition (PECVD) deposition parameters of the SiN*_x_* thin films.

Sample No.	Temperature (°C)	Pressure (mTorr)	SiH_4_ (sccm)	NH_3_ (sccm)	Power (W)
Sample1	200	450	20	10	20
Sample2	200	800	20	10	20
Sample3	100	800	20	10	20
Sample4	100	450	20	10	20
Sample5	100	800	30	20	20

**Table 2.** 2θ value of (002) peak, full width at half maximum (FWHM) values, *E*_g_ values, the average crystallization sizes of the GZO thin films deposited on different substrates and the *V*_oc_ value, *J*_sc_ value, and *F.F.* values of the fabricated amorphous silicon thin film solar cells.

Deposition parameters	100 °C-deposited GZO-PI	100 °C-deposited GZO-SiN*_x_*/PI	200 °C-deposited GZO-PI	200 °C-deposited GZO-SiN*_x_*/PI
Sample abbreviation	Substrate A	Substrate B	Substrate C	Substrate D
2θ value of (002) peak	34.14°	34.16°	34.36°	34.36°
FWHM values	0.360	0.378	0.296	0.293
*E*_g_ values	3.573 eV	3.575 eV	3.623 eV	3.624 eV
crystallization sizes	31.8 nm	27.0 nm	35.2 nm	28.0 nm
*V*_oc_ value	0.790 V	0.805 V	0.785 V	0.790 V
*J*_sc_ value	9.13 mA/cm^2^	8.753 mA/cm^2^	10.13 mA/cm^2^	10.64 mA/cm^2^
*F.F.* value	0.580	0.565	0.588	0.553
